# Suppression of hyperexcitability of trigeminal nociceptive neurons associated with inflammatory hyperalgesia following systemic administration of lutein via inhibition of cyclooxygenase-2 cascade signaling

**DOI:** 10.1186/s12950-018-0200-0

**Published:** 2018-11-26

**Authors:** Yumiko Syoji, Ryota Kobayashi, Nako Miyamura, Tsukasa Hirohara, Yoshiko Kubota, Nobuo Uotsu, Kei Yui, Yoshihito Shimazu, Mamoru Takeda

**Affiliations:** 1 Laboratory of Food and Physiological Sciences, Department of Life and Food Sciences, School of Life and Environmental Sciences, Azabu University, 1-17-71, Fuchinobe, Chuo-ku, Sagamihara, Kanagawa 252-5201 Japan; 2 FANCL Health Science Research Center, Research Institute, FANCL Corporation, 12-13, Kamishinano, Totsuka-ku, Yokohama, Kanagawa 244-0806 Japan

**Keywords:** Inflammation, Edema, lutein, Trigeminal nociceptive neuron, Hyperalgesia, Single-unit recording, cyclooxygenase-2

## Abstract

**Introduction:**

Lutein is a dietary constituent known to inhibit inflammation; however, its effect on nociceptive neuron-associated hyperalgesia remains to be determined. The present study therefore investigated under in vivo conditions whether administration of lutein attenuates the inflammation-induced hyperexcitability of trigeminal spinal nucleus caudalis (SpVc) neurons that is associated with mechanical hyperalgesia.

**Results:**

Complete Freund’s adjuvant (CFA) was injected into the whisker pads of rats to induce inflammation, and then mechanical stimulation was applied to the orofacial area to assess the threshold of escape. The mechanical threshold was significantly lower in inflamed rats compared to uninjected naïve rats, and this lowered threshold was returned to control levels by 3 days after administration of lutein (10 mg/Kg, i.p.) Also the lutein administration, inflammation-induced thickness of edema was returned to control levels. The mean increased number of cyclooxygenase-2 (Cox-2)-immunoreactive cells in the whisker pads of inflamed rats was also returned to control levels by administration with lutein. The mean discharge frequency of SpVc wide-dynamic range (WDR) neurons to both nonnoxious and noxious mechanical stimuli in inflamed rats was significantly decreased after lutein administration. In addition, the increased mean spontaneous discharge of SpVc WDR in inflamed rats was significantly decreased after lutein administration. Similarly, lutein significantly diminished noxious pinch-evoked mean after discharge frequency and occurrence in inflamed rats. Finally, lutein restored the expanded mean size of the receptive field in inflamed rats to control levels.

**Conclusion:**

These results together suggest that administration of lutein attenuates inflammatory hyperalgesia associated with hyperexcitability of nociceptive SpVc WDR neurons via inhibition of the peripheral Cox-2 signaling cascade. These findings support the proposed potential of lutein as a therapeutic agent in complementary alternative medicine strategies for preventing inflammatory mechanical hyperalgesia.

## Introduction

The spinal trigeminal nucleus caudalis (SpVc) is an important relay station for neural trigeminal nociceptive inputs following inflammation and tissue injury [1–3]. SpVc nociceptive neurons are classified as nociceptive-specific (NS) and wide-dynamic range (WDR) based on their sensitivity to mechanical stimulation applied to orofacial areas such as facial skin, with WDR neurons responsive to both noxious and non-noxious stimulation [2]. Since graded noxious stimuli applied to receptive fields produce increased firing frequency of SpVc WDR neurons in proportion to stimulus intensity, it can be assumed that WDR neurons are important for encoding stimulus intensity. Chronic pathological conditions such as tissue inflammation can change the properties of somatic sensory pathways, leading to hyperalgesia [4]. Specifically, inflammation and tissue injury change the excitability of primary afferent neurons (peripheral sensitization), which alters information processing in the trigeminal spinal nucleus or higher centers (central sensitization) [5]. Complete Freund’s adjuvant (CFA) models of inflammation in the orofacial region have been developed in rats to study the trigeminal neural signaling pathways underlying pathological pain [3, 6–8] with CFA inflammation-induced hyperexcitability of SpVc WDR neurons linked to mechanical stimuli [3, 7]. SpVc neurons have also been implicated in the mechanism of hyperalgesia and/or referred pain associated with dental pain [2, 3, 9].

Lutein is a naturally occurring carotenoid present in various food products, such as fruits and leafy green vegetables [10], that inhibits stimulation-induced responses to transient receptor potential ankyrin 1 (TRPA1), but not transient receptor potential vanilloid 1 (TRPV1), on cell bodies and peripheral terminals of sensory neurons in vitro and in vivo [11]. Based on these distinct actions and the carotenoid structure, it was proposed that lutein modulates lipid rafts in the cells membranes around TRP channels [11, 12]. TRPA1 channels also modulate mechanotransduction via the generator potential in primary sensory neurons [13], and lutein was shown to both inhibit acute retinal ischemia via cyclooxygenase-2 (Cox-2) expression [14] and suppress retinal neural damage in vivo during inflammation via the nuclear translocation of nuclear factor (NF-kB) [15, 16]. We also reported recently that chronic dietary administration of the polyphenol resveratrol attenuates inflammation-induced mechanical hyperalgesia and that this effect was mainly due to the suppression of SpVc WDR neuronal hyperexcitability, possibly via inhibition of both the peripheral and central Cox-2 cascade signaling pathways [17]. Indeed, many recent reports have described the use of complementary and alternative medicines (CAM), such as herbal medicines and acupuncture, for the treatment of persistent clinical chronic pain [18], supporting the potential of CAM in preventing trigeminal inflammatory hyperalgesia. Together, these observations strongly suggest that lutein administration attenuates inflammation-induced hyperexcitabilily of the SpVc WDR neurons associated with trigeminal hyperalgesia, and could therefore underlie potential therapeutic agents for preventing inflammatory hyperalgesia, although no studies have addressed this possibility. The present study therefore investigated whether under in vivo conditions, lutein administration attenuates inflammation-induced hyperexcitability of the SpVc neurons associated with hyperalgesia in rats, using behavioral, immunohistochemical, and electrophysiological techniques.

## Methods

The experiments were approved by the Animal Use and Care Committee of Azabu University and were consistent with the ethical guidelines of the International Association for the Study of Pain [19]. Every effort was made to minimize the number of animals used and their suffering. Each experiment was performed such that experimenters were blinded to the experimental conditions.

### Induction of cutaneous inflammation and lutein administration

The experiments were performed on adult male Wistar rats (210–280 g, body weight, *n* = 24). Rats were divided into three groups, as follows: naïve (*n* = 8), inflamed (*n* = 8), and inflamed with lutein (10 mg/kg, i.p.) treatment (*n* = 8), with the dosage based on previous research indicating that 10 mg/kg lutein significantly decreases the inflammatory response [15]. Behavioral study (Escape threshold and thickness of edema) were conducted on 24 rats and 15 out of 24 rats were used on the electrophysiological recording remaining 9 rats were conducted on the immunohistochemical study. Each animal was anesthetized with sodium pentobarbital (45 mg/kg, i.p.), and complete Freund’s adjuvant (CFA) (0.05 ml 1:1 oil/saline suspension) was injected into the left side of the facial skin to induce inflammation, as described previously [3, 17, 20]. For naïve rats, vehicle (0.9% NaCl) alone was injected into the left side of the facial skin.

The lutein (MW = 568.9, Sigma-Aldrich, St-Louise, MO, USA) was dissolved in dimethyl sulfoxide (DMSO), with the stock solution stored at − 20 °C in small aliquots until use. Lutein was administered to rats daily before behavioral, immunohistochemical and electrophysiological experimentation. Based on our results from the behavioral analysis for escape threshold, we conducted immunohistochemical and electrophysiological experiments on days 3. We evaluated the effects of lutein on the symptoms of peripheral inflammation using CFA-induced edema of the whisker pad region and measuring the thickness of edematous areas among the three groups, as described previously [21]. In some experiments, the CFA-induced inflammation was verified with Evan’s blue staining (50 mg/mL, 1 mL/kg, i.v.) extravasation. The accumulation of blue dye in the skin was evaluated on postmortem examination of the injected facial region, indicating that the plasma protein extravasation was due to localized inflammation [3, 6].

### Mechanical threshold for escape behavior

Assessing the mechanical threshold for escape behavior was conducted as described in previous studies [8, 17]. In brief, one and two days after CFA or vehicle injection into the facial skin (the ipsilateral and contralateral facial skin regions were tested), mechanical hyperalgesia was assessed with a set of von Frey hairs (Semmes-Weinstein Monofilaments, North Coast Medical, CA) applied to the whisker pad in ascending series of trials, with three separate von Frey stimulation applied per series. Escape threshold intensity was determined when rats moved their heads away from at least one of the three stimuli.

### Immunohistochemistry of Cox-2 in the whisker pad

Immunohistochemistry was conducted using the modified method described in our previous study [8]. Each rat (*n* = 9) was deeply anaesthetized with pentobarbital sodium (50 mg/kg, i.p.), and transcardially perfused with 50 ml heparinized saline in 0.01 M phosphate-buffered saline (PBS), followed by 100 ml of 4% paraformaldehyde in 0.1 M phosphate buffer (PB, pH 7.3). Tissue from the left side of the whisker pad was removed and incubated progressively in 4% sucrose (3 × 5 min), 10% sucrose (1 h), 20% sucrose (2 h), and then in 30% sucrose overnight. Frozen sections were cut at 10 μm with a cryostat (Leica, Nussloch, Germany) and mounted on silane-coated glass slides. Sections were incubated with rabbit anti-rat Cox-2 polyclonal antibody (1:500, Thermo Scientific, USA) for 15 h at 4 °C, washed in 0.01 M PBS, and then incubated in Alexa® 488 goat anti-rabbit IgG secondary antibody (1:500, Molecular Probes, Eugene, OR). Labeled cryosections were rinsed consecutively in 0.01 M PBS for 5 min each. The samples were mounted with Dapi-Fluoromount-G (Southern Biotech, USA). A control experiment was conducted with primary antibody absorption. The fluorescence intensity difference between the staining with primary antibody omitted and the least intense antibody staining was scored as positive. Every third section was used for immunohistochemistry and 20 sections were analyzed per whisker pad. Cox-2-positive cells were counted in all areas of inflammation in each whisker pad among three groups. The data are presented as mean number of positive cells/mm^2^ and mean percentage of Cox-2-positive cells. Fluorescent images of the stained sections were generated using an Olympus FSX100 all-in-one fluorescent microscope (Olympus, Japan). Digital images were analysed using Olympus Cell Sens microscope imaging software.

### Extracellular single-unit recording of SpVc WDR neuronal activity

Electrophysiological recordings were conducted three days after CFA or vehicle injection. Each animal was first anesthetized with pentobarbital sodium (45 mg/kg, i.p.) and maintained with additional doses of 2–3 mg/kg/h through a cannula into the jugular vein, as required. The level of anesthesia was confirmed by an absence of the corneal reflex and a lack of response to paw pinching. The rectal temperature was maintained at 37 ± 0.5 °C with a homeothermic blanket during recording. All wound margins were continuously covered with a local anesthetic, 2% lidocaine (Xylocaine), throughout the experiments. The animals were then placed in a stereotaxic apparatus, and the activity of a single neuron from the SpVC region was recorded extracellularly using a glass micropipette filled with 2% pontamine sky blue and 0.5 M sodium acetate according to the stereotaxic coordinates of Paxinos and Watson [22]. Neuronal activity was amplified (WPI, DAM 80), filtered (0.3–10 kHz), monitored with an oscilloscope (Iwatsu, SS-7672, Tokyo), and then recorded for off-line analysis by Power Lab and Chart 5 software (ADI Instruments, UK) as described previously [17, 23].

### Experimental protocols

Recordings of the extracellular SpVc WDR unit activity were carried out as follows [17, 23]. Mechanical stimulation (with paint brush) was used as a search stimulus to quickly identify the receptive fields and to avoid sensitization of peripheral receptors. Single unit that responded to left side of orofacial facial skin (whisker pad) with a brush and a set of von Frey hairs was identified. Noxious pinch stimulation was applied to the orofacial area with calibrated forceps for 5 s, based on a level that evoked pain sensation when applied to a human subject [17, 23]. After identifying the SpVc WDR neuronal response in the rat whisker pads, we determined whether there was a spontaneous discharge, and then compared the discharge rates induced by mechanical stimulation between naïve and inflamed rats. The threshold for mechanical stimulation was determined by using non-noxious and noxious mechanical stimulation of von Frey hairs (0.07, 0.16, 0.2, 1, 4, 6, 10, 15, 26, and 60 g) at intervals of 5 s. The mechanical stimulation-receptive neurons were mapped by probing the facial skin with von Frey hairs, and then outlined onto a life-sized drawing of each rat on tracing paper. The SpVc WDR neuronal discharges induced by mechanical stimulation were quantified by subtracting the background activity from the evoked activity. Spontaneous discharge frequencies were determined over 2–5 min. Since previous studies have demonstrated that WDR neurons in the SpVc region have an important role in the mechanism underlying hyperalgesia and referred pain associated with orofacial pain [3, 21, 24], and the focus of the present study was on the effects of lutein on SpVc WDR neuronal activity, we did not examine nociceptive-specific neurons [25]. Peristimulus histograms (bi*n* = 100 ms) were generated in response to each stimulus. After-discharges were recorded for 10 s after the pinching receptive field. Mean spontaneous and mechanical stimulation-evoked discharges frequencies, after-discharge frequencies, and mean mechanical thresholds of SpVc WDR neurons were compared among three groups (naïve, CFA, and CFA rat with lutein).

### Identification of recording site

At the end of recording sessions, rats were deeply anesthetized, and anodal DC currents (30 μA, 5 min) were passed through a recording micropipette. The animals were transcardially perfused with saline and 10% formalin. Coronal sections were cut frozen at 30 μm, thawed, and then stained with hematoxylin-eosin. Recording sites were identified from the blue spots, and construction of the electrode tracks was done by combination with the micromanipulator readings.

### Data analysis

Values are expressed as means ± SEM. Statistical analysis was performed using one-way repeated measure analysis of variances (ANOVAs) followed by the Tukey-Kramer/Dunnett’s tests (post hoc test) for behavioral and electrophysiological data. *P* < 0.05 was considered statistically significant.

## Results

### Induction of inflammation-induced hyperalgesia

**Fig. 1:**
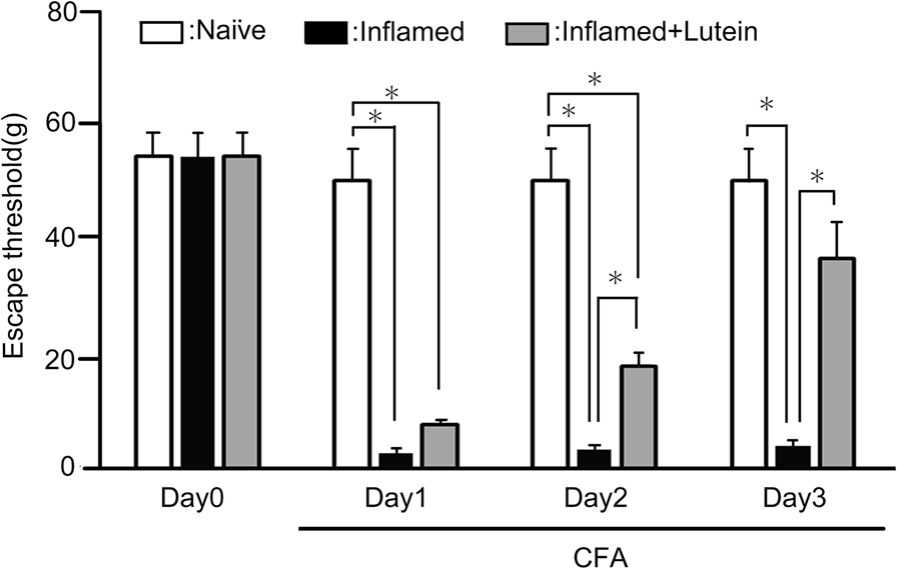
Comparison of change in the escape threshold among naïve, inflamed, and inflamed with lutein administration rats. Mechanical stimulation using von Frey hairs was applied to the ipsilateral whisker pad of naïve (saline), complete Freund’s adjuvant (CFA)-inflamed, and CFA-inflamed with lutein (10 mg/kg, i.p) rats to assess hyperalgesia. Data are mean ± SEM. *P* < 0.05, naïve (*n* = 8) vs. CFA (*n* = 8), CFA vs. CFA with lutein (*n* = 8)

In this study, after CFA injection into the whisker pad, the rats were tested for hyperalgesia by probing the injected site and/or the orofacial skin with von Frey filaments. In inflamed rats, CFA significantly reduced the threshold for escape from mechanical stimulation applied to the whisker pad area from 53.2 ± 3.8 g in naïve rats to 2.4 ± 1.5 g at 3 days after the injection (*n* = 8, *P* < 0.05; Fig. 1). No significant changes in the contralateral threshold in the whisker pad area were observed between two groups (naïve vs. inflamed; 68.1 ± 3.8 g vs. 59.3 ± 4.2 g, *n* = 8, NS).

### Administration of lutein attenuates hyperalgesia

Following daily lutein administration, the reduced escape threshold from mechanical stimulation in day 2 inflamed rats was partially returned to control levels, but still significantly at low level, compared to naïve rats (Fig. 1). As shown in Fig. 1, the reduced escape threshold from mechanical stimulation in inflamed rats was significantly returned to control level by administration of lutein at 3 days after inflammation (naive vs. day 3 inflamed with lutein; 53.2 ± 15.2 g vs. 37.3 ± 6.7 g, *n* = 8, NS).

### Administration of lutein inhibits inflammation-induced edema

**Fig. 2:**
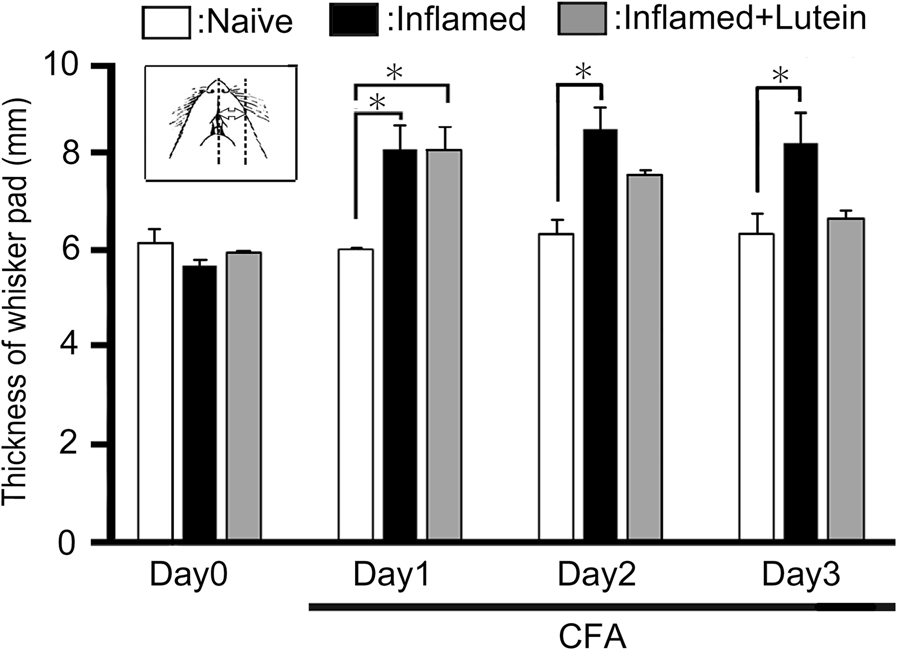
Comparison of change in CFA-inflammatory edema among naïve, inflamed, and inflamed with lutein administration rats. Each column shows the mean thickness of the whisker pad edematous area among three groups of rats. *, *P* < 0.05, naïve vs. inflamed, inflamed vs. inflamed at 3 days before lutein (10 mg/kg, i.p.) treatment, *n* = 8. Inset: Region for the measurement of CFA-induced orofacial inflammatory edematous area

The edematous area of whisker pad in the inflamed rats was increased in thickness compared to that in naïve rats, from one day to three days (*P* < 0.05), with the day 2 thickness of naïve vs. inflamed at 6.0 ± 0.1 mm vs. 8.1 ± 0.5 mm, *n* = 8 (Fig. 2). Two and three days after the lutein administration, the mean thickness of edema was returned to control levels (naïve vs. inflamed at 3 days with lutein, 6.3 ± 0.4 mm vs. 6.7 ± 0.1 mm, *n* = 8, NS) (Fig. 2).

### Lutein administration inhibits Cox-2 immunoreactivity in the whisker pad

**Fig. 3:**
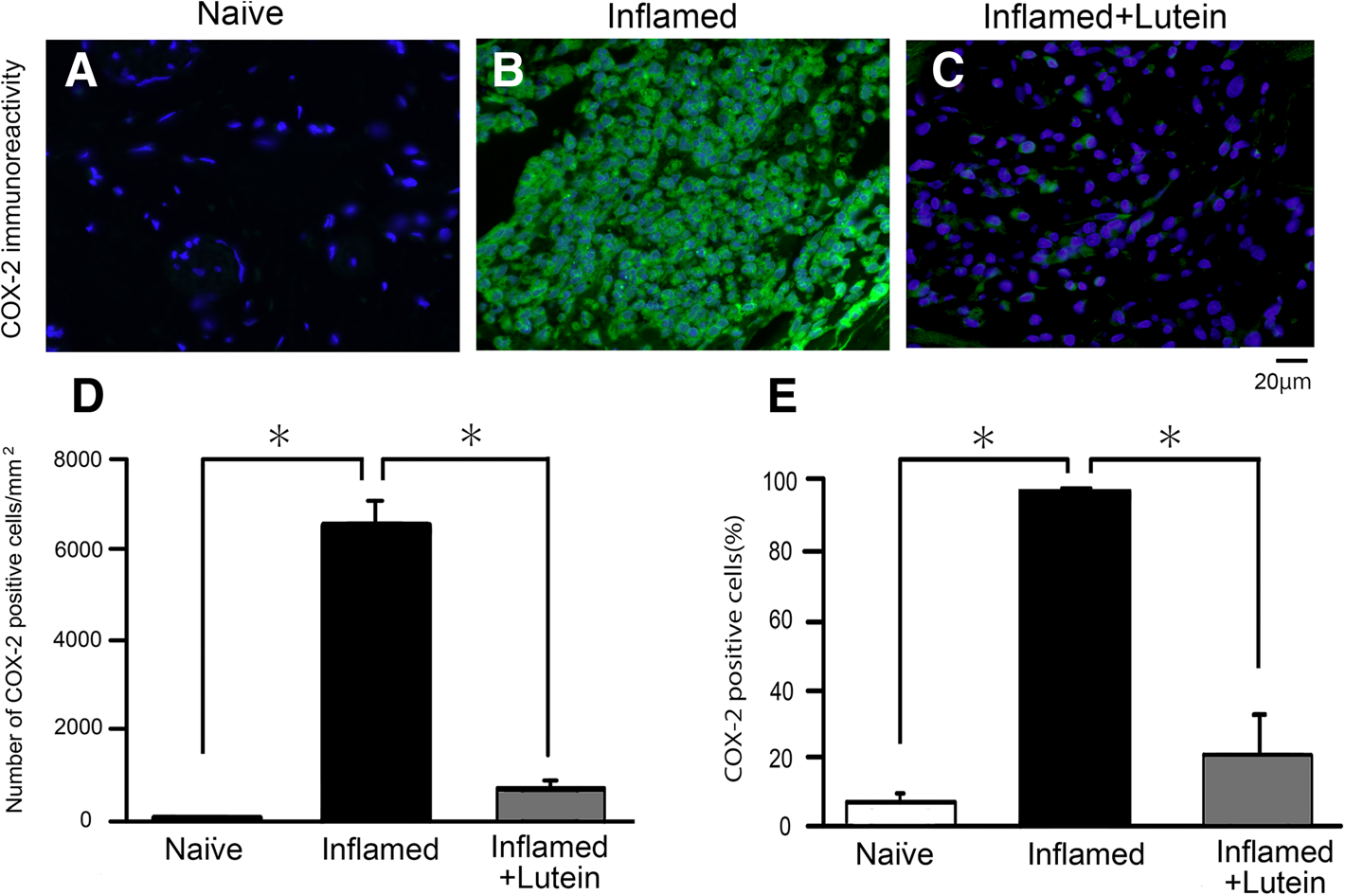
Lutein pre-treatment attenuates the number of Cox-2-immunoreactive cells in whisker pad among naïve, inflamed, and inflamed with lutein administration rats. **a** Naïve rats, **b** CFA-induced inflamed rats, **c** Inflamed rats 3 days after treatment (10 mg/kg, i.p.). **d** Comparison of the number of Cox-2-immunoreactive cells in the orofacial skin among three groups of rats. *, *P* < 0.05, naïve vs. inflamed rats and inflamed vs. inflamed with lutein. **e** Comparison of the mean percentage of Cox-2-immunoreactive cells in the orofacial skin among three groups of rats. *, *P* < 0.05, naïve vs. inflamed rats and inflamed vs. inflamed with lutein, *n* = 3

Figure 3a represents a typical example of Cox-2-immunoreactive cells in the whisker pad area of naïve (*n* = 3), inflamed for 3 days (*n* = 3) and inflamed with lutein administration day 3 rats (*n* = 3). As shown in Fig. 3a, b and c, following injection of CFA into the whisker pads, the mean number of Cox-2*-*immunoreactive cells in the whisker pad was significantly increased in the inflamed rats compared to the similar areas of naïve rats (69.0 ± 25.5/mm^2^ vs. 6566.9 ± 518.4/mm^2^, *P* < 0.05). As shown in Fig. 3d and e, the increased percentage of Cox-2*-*immunoreactive cells and the mean cell number in inflamed rats was returned to control levels by chronic administration of lutein at day 3 of inflammation (inflamed vs. inflamed with lutein; 97.9 ± 0.8% and 6566.9 ± 518.4/mm^2^ vs. 21.3 ± 11.9% and 690.7 ± 168.5/mm^2^, respectively, *n* = 3, *P* < 0.05). In the absence of primary antibody, only background staining was evident (data not shown).

### General characteristics of SpVc WDR neurons

**Fig. 4:**
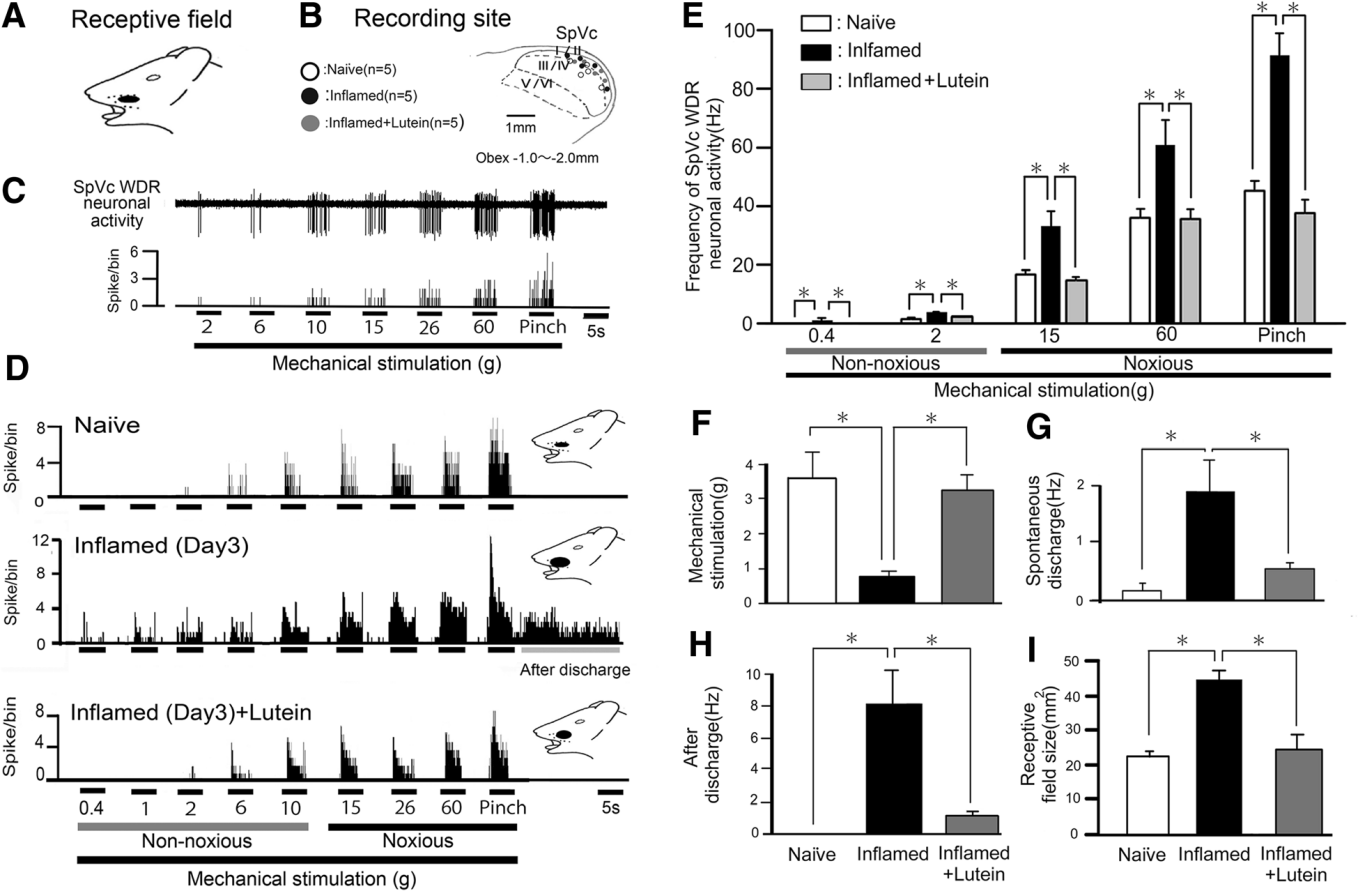
Reversal by daily lutein administration of SpVc WDR neuronal hyperactivity after CFA induction of inflammation. a-c General characteristics of spinal trigeminal nucleus caudalis (SpVc) wide-dynamic range (WDR) neuronal activity responses in orofacial skin. **a** Receptive field of whisker pad in the facial skin. **b** Distribution of SpVc WDR neurons responding to non-noxious and noxious mechanical stimulation of facial skin (*n* = 15). The number below each drawing indicates the frontal plane in relation to the obex. **c** Typical example of non-noxious and noxious mechanical stimulation-induced firing of SpVc WDR neurons. **d** Lutein administration reversed the hyperactivity of SpVc WDR neuronal activity after orofacial CFA inflammation. Example of non-noxious and noxious mechanical stimulation-induced discharge of SpVc WDR neurons in naïve, inflamed, and inflamed with lutein administration (10 mg/kg, i.p.) rats. Note that the decreased mechanical stimulation threshold needed to evoke neuronal firing, increased spontaneous discharges, increased size of receptive field, and occurrence of noxious pinch-evoked discharges in the inflamed rats were reversed to control levels following lutein administration. **e** Comparison of mean discharge frequency of SpVc WDR neurons evoked by mechanical stimulation (non-noxious and noxious) of whisker pad among three groups of rats. *, *P* < 0.05, naïve vs. inflamed rats and inflamed vs. inflamed with lutein *, *P* < 0.05, naïve vs. inflamed rats, inflamed vs. inflamed with lutein. **f** Comparison of mean mechanical thresholds of SpVc WDR neurons among three groups of rats. *, *P* < 0.05, naïve vs. inflamed rats and inflamed vs. inflamed with lutein. **g** Comparison of mean spontaneous discharges of SpVc WDR neurons among three groups of rats. *, *P* < 0.05, naïve vs. inflamed rats and inflamed vs. inflamed with lutein. **h** Comparison of mean noxious pinch-evoked after-discharge frequencies of SpVc WDR neurons among three groups of rats. *, *P* < 0.05, naïve vs. inflamed rats and inflamed vs. inflamed with lutein. **i** Comparison of mean receptive field size of SpVc WDR neurons among three groups of rats. *, *P* < 0.05, naïve vs. inflamed rats and inflamed vs. inflamed with lutein

A total of 15 SpVc WDR neurons responding to mechanical stimulation of whisker pad were analyzed in naïve (*n* = 5), inflamed (*n* = 5), and inflamed rats with lutein administration (*n* = 5). These SpVc neurons responding to non-noxious and noxious mechanical stimulation exhibited a somatic receptive field in the orofacial area (mainly whisker pad) (Fig. 4a). Every neuron recorded belonged to the category of WDR neurons, and the typical recording sites were mainly distributed in the maxillary and mandibular branches (Fig. 4b). As shown in Fig. 4b, recording sites were found in layers I- III (*n* = 9, 60%) and IV-V (*n* = 6, 40%) in the SpVc (obex − 1.0~ − 2.0 mm). There were no obvious differences in recording site with each unit among the three groups. As shown in Fig. 4c, graded mechanical stimulation applied to the most sensitive area of receptive field showed increased firing frequency of SpVc neurons as proportional to stimulus intensity, as described previously [3, 17, 26], with a mean mechanical stimulation induced spike threshold of 3.6 ± 0.3 g. Every neuron recorded belonged to the category of WDR neurons.

### Changes in excitability of SpVc WDR neurons following inflammation

We confirmed in this study that CFA induced hyperexcitability of SpVc WDR neurons, as described in our previous study [17, 26]. In naïve rats, spontaneous discharges were observed in 20% (1/5) of SpVc neurons (Fig. 4d); most neurons fired at a low frequency and the mean firing frequency was 0.1 ± 0.2 Hz (*n* = 5), whereas all of the WDR neurons (5/5;1.9 ± 0.6 Hz) were spontaneously active in inflamed rats (Fig. 4d). In inflamed rats, SpVc WDR neurons showed significantly stronger responses to non-noxious mechanical stimulation compared with naïve rats (Fig. 4d), as described in a previous study [17, 27]. The mean firing frequencies of SpVc WDR neurons in response to mechanical stimuli (0.4, 2, 15, 60 g, pinch) were also significantly greater in inflamed rats than in control rats (*n* = 5; Fig. 4e), and the mean mechanical threshold in inflamed rats was significantly decreased to 0.8 ± 0.1 g compared with 3.6 ± 0.7 g in naïve rats (*n* = 5; Fig. 4d and f), while the mean spontaneous discharge frequency of inflamed rats was significantly increased compared to that of naïve rats (Fig. 4d and g). Although no obvious noxious pinch-evoked after-discharges were recorded in naïve rats (0/5), most of SpVc neurons in inflamed rat (5/5:100%) showed after-discharges following noxious pinch stimulation (frequency; 8.1 ± 2.2 Hz, duration; 21.2 ± 6.2 s) (Fig. 4d and h). The mean receptive size in the inflamed rats was significantly increased to 44.6 ± 1.9 mm^2^ compared to 22.4 ± 2.0 mm^2^ (*n* = 7, *P* < 0.05).

### Lutein administration inhibits hyperexcitability of SpVc WDR neurons in inflamed rats

According to our results obtained from the behavioral analysis for escape threshold, we tested the effect of chronic administration of lutein on the hyperexcitability of SpVc WDR neurons in inflamed day 3 rats. Typical examples of lutein administration on the discharge rates of SpVc WDR neurons responding to non-noxious (0.07–10 g) and noxious mechanical (15–60 g, pinch) stimulation in inflamed rats are shown in Fig. 4d. After chronic daily administration of lutein over three days in inflamed rats, discharge frequency of SpVc WDR neurons to both non-noxious and noxious mechanical stimulation were decreased to the control level (Fig. 4d), and the lowered mechanical threshold and augmented spontaneous and noxious and non-noxious firing frequencies in inflamed rats were returned to those observed in naïve rats. As shown in Fig. 4e, the mean number of discharge frequency of SpVc WDR neurons in inflamed rats was significantly decreased after lutein administration for both non-noxious and noxious mechanical stimuli (*P* < 0.05). The mean mechanical stimulation threshold in inflamed rats after lutein was also significantly reversed to control levels (Fig. 4f). Spontaneous discharges of SpVc WDR neurons in inflamed rats were also significantly decreased after lutein administration (Fig. 4g, *P* < 0.05). The noxious pinch-evoked after-discharge frequency in inflamed rats was decreased after lutein administration (inflamed vs. inflamed with lutein: 100% vs. 40%). Indeed, the mean noxious pinch-evoked after-discharge firing frequency in inflamed rats was significantly decreased to control levels (Fig. 4h), as was the mean size of receptive field in inflamed rats (Fig. 4i). Chronic vehicle administration had no significant effect on spontaneous and non-noxious, noxious mechanical, or pinch stimulation-evoked hyperexcitability of SpVc WDR neuron in inflamed rats (data not shown).

## Discussion

### Administration of lutein attenuates trigeminal inflammatory hyperalgesia

The present study produced the following main findings: *(i)* the threshold of escape from mechanical stimulation applied to the orofacial area in inflamed rats was significantly lower than that in naïve rats, as described previously [8, 17]; *(ii)* from one to three days after inflammation, the lowered mechanical threshold in the inflamed rats tended toward control levels following daily administration of lutein (10 mg/kg, i.p); *(iii)* the reduced escape threshold from mechanical stimulation in inflamed rats increased to control levels with lutein at day 3 of inflammation; *(iv)* inflammation-induced edema was significantly decreased to control levels with lutein at 2 days inflammation.

Matsumoto et al. [21] recently reported that pretreatment with resveratrol significantly decreased the mean thickness of inflammation-induced edema in whisker pads compared to those of untreated, inflamed rats and significantly decreased that number of *c-fos*-immunoreactive SpVc/C1 neurons in inflamed rats compared to naïve rats. Recently, we also reported that administration of dietary resveratrol attenuates inflammation-induced mechanical inflammatory hyperalgesia and that this effect was mainly due to the suppression of hyperexcitability of SpVc WDR neurons via the inhibition of both peripheral and central Cox-2 cascade signaling pathways [17]. In accordance with these observations, the present study revealed that *(i)* following injection of CFA into the whisker pads, the mean number of Cox-2*-*immunoreactive cells in the whisker pad was significantly increased in the inflamed rats compared to those in the naïve rats; *(ii)* increased Cox-2 immunoreactivity of whisker pad in inflamed rats was significantly returned to control level by administration of lutein day 3. Taken together, these findings support the idea that daily administration of lutein suppresses inflammation-induced edema and hyperalgesia via the inhibition of prostaglandin E_2_ (PGE_2_) production by suppression of Cox-2 signaling in the whisker pad.

### Suppressive effect of lutein on the hyperexcitability of WDR SpVc neuronal activity associated with hyperalgesia following inflammation

Scholz and Woolf [4] indicated that peripheral tissue injury/inflammation innervating trigeminal nerves can alter the properties of trigeminal somatic sensory pathways, causing behavioral hypersensitivity and resulting in increased responses to pain caused by noxious stimulation, such as hyperalgesia. Following peripheral inflammation and/or nerve injury, inflammatory mediators such as PGE_2_ bind to G-protein-coupled E-type prostanoid receptors and induce activation of protein kinases A and C (PKA and PKC, respectively) in nociceptive peripheral terminals, leading to phosphorylation of mechanosensitive sodium and potassium ion channels and receptors [26, 27]. As a result, the activation threshold for transducer channels such as TRPA1 is reduced and the membrane excitability of the peripheral terminals increases, resulting in a high frequency of action potentials being conducted to presynaptic central terminals of the SpVc [2, 26]. This induces the release of large amounts of glutamate into the synaptic cleft, which binds to upregulated post-synaptic glutamate receptors, augmenting excitatory post synaptic potentials (EPSPs), causing a barrage of action potentials to be conducted to higher centers of pain pathways, and creating a state of heightened sensitivity termed peripheral sensitization [2, 26].

In this study, we found that the decreased mean mechanical stimulation threshold in the inflamed rats was also significantly returned to control levels following daily systemic administration of lutein. In accordance with these findings, both non-noxious and noxious mechanical stimuli-evoked mean discharge frequencies of the SpVc WDR neurons in inflamed rats were significantly returned to control levels after chronic lutein treatment, suggesting that systemic administration of lutein can alter the inflammation-induced hypersensitivity of SpVc WDR neuronal activity, possibly via the suppression of peripheral sensitization. The present study also revealed that lutein administration returned the increased Cox-2*-*immunoreactivity of whisker pad in inflamed rats to control levels. To this end, PGE_2_ appears to facilitate the activation of TRPA1 and tetrodotoxin-TTX-resistant (TTX-R) Na^+^ channels [4, 28, 29], with the latter channels (eg., Nav1.8 and Nav1.9) selectively expressed in small- and medium-sized DRG neurons [30]. Based on findings of increased excitability of small-diameter trigeminal ganglion neurons after PGE_2_ application and an increase in the TTX-R Na^+^ currents [31], it can be assumed that lutein inhibits the excitability of small-diameter trigeminal ganglion neurons via suppression of Cox-2-related PGE_2_ production leading to induced generator potential and TTX-R Na^+^ currents.

Previous studies reported after-discharges followed by noxious mechanical stimulation in SpVc WDR neurons tested in a chronic inflammation model, in association with neuronal sensitization during persistent pain [32, 33]. Interestingly, in this study, we observed that after-discharges following the noxious pinch stimulation in inflamed rats were abolished following daily administration of lutein. Although the precise mechanism underlying this suppressive effect was not elucidated, two possible mechanisms can be postulated. First, previous administration of the substance P receptor, neurokin-1 (NK_1_) receptor antagonist inhibited pinch-evoked after-discharges of WDR neurons in the spinal cord [34]. We also have previously observed the evidence that CFA-induced pinch-evoked after-discharges of SpVc WDR neurons was abolished by resveratrol administration [17]. Thus, although there are no reports for lutein acting as a NK1 receptor antagonist, it can be assumed that chronic administration of lutein could similarly attenuate NK1 receptor-mediated after-discharges of WDR neurons in the SpVc under inflammatory conditions. In a second series of data, microiontophoretic application of GABA_A_ receptor agonists/antagonists into the SpVc region using multibarrel electrodes suggested that a local GABAergic mechanism could control nociceptive transmission in the SpVc neurons, contributing to the overall mechanical receptive field properties [24]. Since Higashima et al. [35] also reported that GABA_A_ receptor antagonist, bicuculline, inhibits generation of after-discharges from hippocampal neurons, while GABA_B_ receptor antagonist, phaclofen, enhances after-discharges in slice preparations, it can be assumed that chronic administration of lutein attenuates GABA_A_ receptor-mediated after-discharges of WDR neuron in the SpVc. In this study, we also found that the mean receptive field size of inflamed rats was returned to control levels following lutein. Although the precise mechanism by which chronic administration of lutein could suppress the expanded receptive field remains to be understood, it can be postulated that lutein modulates a local GABAergic mechanism tonic control and that nociceptive mechanoreceptive transmission predominantly inhibits central mechanisms through excitatory synaptic transmission. It is clear that further studies are warranted to explore this intriguing possibility, but it is reasonable to speculate that at least part of the peripheral anitinociceptive action of lutein is due to prevention of peripheral sensitization, as well as antipyretic analgesics.

### Functional significance of suppression effect of lutein on the hyperexcitability of SpVc neuron associated with hyperalgesia

In this study, spontaneous discharges were observed in 20% of naïve rats, while most neurons fired at a low frequency and all SpVc WDR neurons were spontaneously active in inflamed rats. Previous studies reported that CFA inflammation induced hyperexcitability of SpVc WDR neurons to mechanical stimuli [3, 17], and there are reports that SpVc WDR neurons contribute to the mechanism of hyperalgesia and/or referred pain associated with dental pain [3, 20], while ongoing activities were observed in the SpVc associated with ongoing headache and spontaneous pain [36]. Indeed, the origin of ongoing activity in the central neurons that relay sensory information has become a topic of considerable clinical interest because of findings suggesting such activity as a quantitative determinant of post-traumatic injury and chronic pain [36, 37]. To this end, Roch et al. [37] demonstrated that ongoing activity of SpVc WDR neurons is driven from the periphery, since microinjection of lidocaine into the trigeminal ganglia caused a significant decrease in ongoing activity. Taken together with these results, our data suggest that lutein could suppress spontaneous discharge activity of SpVc WDR neurons innervating the facial skin due to peripheral and/or trigeminal ganglion sensitization [9, 37], with probable contributing effects on spontaneous pain, including clinical headache.

The toxic side effects associated with most commonly prescribed analgesic drugs, such as NSAIDs, cox-inhibitors, and opioids, have increased interest in CAM agents for the treatment of persistent chronic pain [38, 39]. Indeed, patients frequently turn to CAM therapies including herbal medicines and acupuncture for pain control when other medical treatments become ineffective [18, 39], and the potential influence of diet and dietary supplementation on conditions associated with pain are increasingly the focus of research [40–42]. In the present study, we found that systemic administration of lutein attenuated inflammation-induced hyperexcitability of trigeminal SpVc neurons associated with hyperalgesia in rats. Sekiguchi et al. [17] also reported that chronic administration of resveratrol attenuates inflammation-induced mechanical inflammatory hyperalgesia, mainly via suppression of SpVc WDR neurons and inhibition of both peripheral and central Cox-2 signaling pathways. Therefore, our results combined with this finding suggest that dietary constituents such as lutein and resveratrol might additively contribute to the development of analgesic drugs with fewer and less toxic side effects for treating pathological pain, including orofacial pain. In particular, our findings support that lutein is a potential therapeutic alternative for clinical use in preventing trigeminal inflammatory hyperalgesia.

## Conclusion

The present study provided the first evidence that administration of lutein attenuates inflammatory hyperalgesia associated with hyperexcitability of nociceptive SpVc WDR neurons via the inhibition of peripheral Cox-2 signaling. These findings support the idea that lutein is a potential therapeutic agent for preventing trigeminal inflammatory hyperalgesia.
